# Influence of health-insurance on treatment outcome of childhood cancer in Western Kenya

**DOI:** 10.1007/s00520-023-07913-1

**Published:** 2023-07-15

**Authors:** Sandra Langat, Festus Njuguna, Gilbert Olbara, Hugo Martijn, Cenne Sieben, Moniek Haverkort, Dennis Njenga, Terry A. Vik, Gertjan Kaspers, Saskia Mostert

**Affiliations:** 1 Academic Model Providing Access to Healthcare (AMPATH), Eldoret, Kenya; 2 Emma’s Children Hospital, Amsterdam UMC, Vrije Universiteit, Amsterdam, The Netherlands; 3 Department of Child Health and Pediatrics, Moi Teaching and Referral Hospital, Moi University, Eldoret, Kenya; 4 Department of Pediatrics, Division of Hematology-Oncology, Indiana University School of Medicine, Indianapolis, IN USA; 5 Princess Máxima Center for Pediatric Oncology, Utrecht, The Netherlands

**Keywords:** Childhood cancer, Health-insurance, Low and middle-income countries

## Abstract

**Background:**

Few governments in low and middle-income countries (LMIC) have responded favourably to the international plea for Universal Health Coverage. Childhood cancer survival in LMIC is often below 20%. Limited health-insurance coverage may contribute to this poor survival. Our study explores the influence of health-insurance status on childhood cancer treatment outcomes in a Kenyan academic hospital.

**Methods:**

This was a retrospective medical records review of all children diagnosed with cancer at Moi Teaching and Referral Hospital between 2010 and 2016. Socio-demographic and clinical data was collected using a structured data collection form. Fisher’s exact test, chi-squared test, Kaplan–Meier method, log-rank test and Cox proportional hazard model were used to evaluate relationships between treatment outcomes and patient characteristics. Study was approved by Institutional Research Ethics Committee.

**Findings:**

From 2010–2016**,** 879 children were newly diagnosed with cancer. Among 763 patients whose records were available, 28% abandoned treatment, 23% died and 17% had progressive/relapsed disease resulting in 32% event-free survival. In total 280 patients (37%) had health-insurance at diagnosis. After active enrolment during treatment, total health-insurance registration level reached 579 patients (76%). Treatment outcomes differed by health-insurance status (*P* < 0.001). The most likely treatment outcome in uninsured patients was death (49%), whereas in those with health-insurance at diagnosis and those who enrolled during treatment it was event-free survival (36% and 41% respectively). Overall survival (*P* < 0.001) and event-free survival (*P* < 0.001) were higher for insured versus uninsured patients. The hazard-ratio for treatment failure was 0.30 (95% CI:0.22–0.39; *P* < 0.001) for patients insured at diagnosis and 0.32 (95% CI:0.24–0.41; *P* < 0.001) for patients insured during treatment in relation to those without insurance.

**Interpretation:**

Our study highlights the need for Universal Health Coverage in LMIC. Children without health-insurance had significantly lower survival. Childhood cancer treatment outcomes can be ameliorated by strategies that improve health-insurance access.

## Introduction

The World Health Organization, the United Nations and over 500 health and development organizations worldwide have called upon governments from low and middle-income countries to effect Universal Health Coverage (UHC) thereby improving access to healthcare services for their citizens. Until now only a few government leaders in low and middle-income countries have responded favourably to this international plea [1].

UHC has the potential of transforming health systems especially for the poorest people. It safeguards people from being pushed into poverty due to out-of-pocket health expenditure by ensuring they can access health services. They need such an access to keep them healthy and productive [2, 3].

An estimated 429,000 children in the world are diagnosed with cancer each year of whom 90% live in low and middle-income countries where there is poor access to healthcare [4]. Survival rates in childhood cancers differ broadly by region. High-income countries may surpass 80% survival, whereas in low and middle-income countries it ranges from 10 to 50%. This poor survival is importantly due to poverty-related barriers in accessing care and treatment abandonment [5, 6].

High costs, limited financial resources and lack of health insurance are among the issues that make diagnosis and treatment of childhood cancer in low and middle-income countries challenging. Out-of-pocket medical expenses negatively affect the treatment outcomes and quality of life of cancer patients as well as being a source of distress for families. These expenses have long-term effects on the financial well-being of the whole family [7–9]. Health-insurance access may be inadequate for the poor majority in low and middle-income countries and lack of it may cause delays in health-seeking behaviour resulting in advanced disease stages at diagnosis, treatment abandonment and worse treatment outcomes [10, 11].

Nearly all African countries have incorporated UHC as a goal in their national health strategies but transforming this into equitable and quality health services and increased financial protection has been slow [12]. The Kenyan government has also invested to make health services more effective, accessible and affordable but access to basic health services through health-insurance coverage remains to be a significant challenge. The National Hospital Insurance Fund (NHIF) was established by the Kenyan government in 1966. NHIF membership is open to all Kenyans who have attained the age of 18 years. Contribution for self-employed and those in the informal sector is around 5 Euro per month whereas contributions for those in formal employment are based on income. New members have to wait for 60 days before the card matures. NHIF covers in-patient and out-patient services in public hospitals and selected private hospitals for one contributor and nuclear family members [13]. Although many families without health-insurance coverage are pushed into abject poverty and inhumane treatments, such as detention in hospitals for their inability to pay medical bills or dispose of valuable family assets, only a minority of Kenyans are registered as members of the affordable NHIF [14–16]. Having health-insurance could have the potential of improving treatment outcomes and survival [12, 13].

There is a strong need to gain insight into the impact of health-insurance on childhood cancer treatment outcomes in low and middle-income countries. This study aims to assess the overall treatment outcomes of children diagnosed with cancer at a Kenyan academic hospital and to evaluate the influence of their health-insurance status (being either uninsured, insured at diagnosis, or enrolled in health-insurance during treatment) on treatment outcomes.

## Methods

### Setting

Kenya is a lower middle-income country in East Africa, bordering the Indian Ocean, between Somalia and Tanzania. Its total population is estimated at 47 million people of which 39% (18 million) are children aged 0–14 years [17].

This study was conducted at Moi Teaching and Referral Hospital (MTRH) in Eldoret, a city in Western Kenya. MTRH serves an estimated population of 24 million people. Childhood cancer incidence is estimated at 15.3 per 100,000 per year. The expected number of children with cancer in the MTRH catchment area would therefore be 1,350. The hospital has 72 pediatric beds, of which 16 are reserved for oncology patients. Nearly all beds are always occupied by more than one patient [18–20]. The paediatric oncology unit is supervised by two paediatricians. Treatment options for patients with cancer include surgery and chemotherapy, but radiotherapy was not available until recently. If radiotherapy was required, children with cancer could be referred to one public hospital in Nairobi at the Kenyatta National Hospital and several private hospitals both in Eldoret and Nairobi, the latter being costly.

At MTRH, dedicated medical personnel assist uninsured parents of children diagnosed with cancer with the health-insurance enrolment process. At the same time the medical team in the unit including clinicians, nurses, and nutritionists talks to families about the importance of registration.

### Study design

This was a retrospective medical records review. Inclusion criteria were: all children between 0 and 16 years of age, who were newly diagnosed with cancer at MTRH between January 2010 and December 2016. Further selection of patients did not take place.

Socio-demographic and clinical data were obtained from the medical records using a structured data collection form. Socio-demographic characteristics including age at diagnosis, gender, patients’ residence, distance to hospital, and health-insurance status were collected. Clinical characteristics included type of cancer, date of diagnosis, duration of symptoms prior to hospital admission at MTRH (< 1 month, 1–3 months, > 3 months), date of start treatment, and treatment outcomes. Representative data from lab, pathology and radiology reports were extracted. Malignancies were classified in 3 groups: 1) haematological tumours; 2) solid tumours; 3) neuro-oncology tumours.

Treatment outcome was defined as either first treatment failure that occurred (abandonment of treatment, death, progressive or relapsed disease), or in case no treatment failure occurred as event-free survival. Treatment abandonment is defined as failure to start or sustain treatment during four or more consecutive weeks and is the most severe form of non-adherence [21].

Health-insurance status was determined by whether: 1) patients had health-insurance at time of diagnosis, 2) patients were enrolled into health-insurance during treatment, or 3) patients never had health-insurance. This data is routinely recorded in patients’ medical records at MTRH.

Our study was approved by MTRH’s Institutional Research Ethics Committee (0003023) and was performed in line with the principles of the Declaration of Helsinki.

Our study being a minimal risk, retrospective file review study, we requested for a waiver of the informed consent.

### Data analysis

Data management and analysis was performed using Excel, SPSS version 23 and R Studio version 4.2.2. The relationship between treatment results and patient (socio-demographic and clinical) characteristics were evaluated by the chi-squared test and Fisher’s exact test. The probability of overall survival and event-free survival was estimated by the method of Kaplan and Meier; estimates were compared using the log-rank test. Overall survival was measured from the date when the patient received a diagnosis of a malignancy to the date of death or the date of last follow-up. Event-free survival was measured from the date of diagnosis to the first treatment failure (abandonment of treatment, death, progressive or relapsed disease) or the date of last follow-up. Cox proportional hazards regression was used to evaluate the effect of patient characteristics on the risk of treatment failure. The analysis was performed using R Studio version 4.2.2. To assess the proportional hazards assumption, Schoenfeld residuals were examined graphically and tested for independence of the covariate by plotting them against time. Variables with non-proportional hazards (violated the proportional hazards assumption) were accounted for by stratifying the analysis or by including interaction terms. Model adjustment variables were chosen a priori based on clinical relevance, and included age at diagnosis, gender, patients’ residence, distance to hospital, health-insurance status, type of cancer, duration of symptoms prior to hospital admission, and date of start treatment. A P-value of less than 0.05 is considered statistically significant.

## Results

**Fig. 1:**
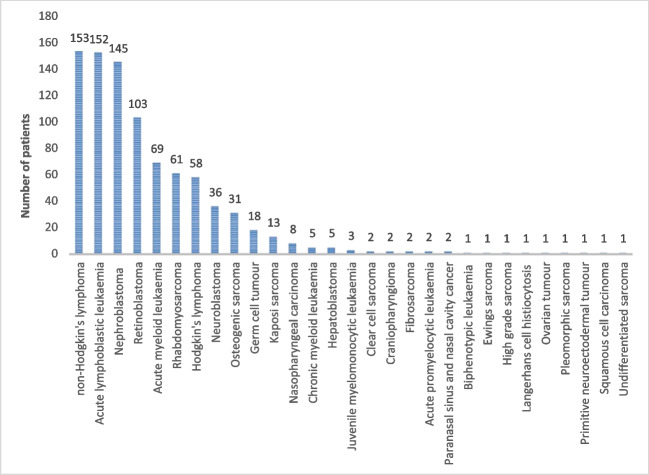
Distribution of different types of cancer diagnosed at MTRH between January 2010 and December 2016 (*n* = 879)

From January 2010 until December 2016, 879 children were consecutively newly diagnosed with a malignancy at MTRH. The number of new childhood cancer patients ranged from 87 (2010) to 161 (2016) with a yearly average of 126 patients. Table 1 lists the sociodemographic and clinical characteristics of these children. Of all 879 patients, 57% were boys. Patients’ age at diagnosis ranged between 0 and 16 years old with a mean of 6.5 years. Malignancies were classified as: haematological tumours (50%), solid tumours (38%), and neuro-oncology tumours (12%). Figure 1 shows the distribution of the different types of childhood cancer diagnosed at MTRH. The top three cancers were non-Hodgkin’s lymphoma (17%), acute lymphoblastic leukaemia (17%) and nephroblastoma (16%). Of the 879 patients with confirmed diagnosis, medical records for 116 (13%) were missing and excluded from further analysis. No significant differences in gender, age, and type of tumour were found between the group of patients with (*n* = 763) or without (*n* = 116) available file.

**Table 1:** Sociodemographic and clinical characteristics of 879 children diagnosed with cancer at MTRH between January 2010 and December 2016

Characteristics	Number of patients n (%)	Health-insurance status		
		No health-insurance	Health-insurance at diagnosis	Health-insurance enrolment during treatment
Gender (*n* = 879)				
Male	505 (57%)	113 (61%)	213 (76%)	179 (60%)
Female	374 (43%)	71 (39%)	67 (24%)	236 (40%)
Age in Years (*n* = 879)				
Mean ± SD (range)	6.5 ± 3.9 (0–16)	6.3 ± 3.9 (0–16)	6.5 ± 3.8 (0–16)	6.7 ± 4.1 (0–16)
Classification of malignancies (*n* = 879)				
Haematological tumours Solid tumours	444 (50%) 330 (38%)	97 (53%) 63 (34%)	174 (62%) 8 (24%)	173 (58%) 199 (7%)
Neuro-oncology tumours	105 (12%)	24 (13%)	38 (14%)	43 (15%)
Distance to MTRH (*n* = 761)				
< 50 km	94 (12%)	27 (15%)	36 (13%)	31 (10%)
50–100 km	204 (27%)	46 (25%)	88 (31%)	70 (23%)
> 100 km	463 (61%)	111 (60%)	141 (50%)	341 (67%)
Referred from other facility (*n* = 730)				
Yes	651 (89%)	156 (85%)	277 (99%)	218 (73%)
No	79 (11%)	28 (15%)	3 (1%)	81 (27%)
Diagnosed with cancer at other facility (*n* = 648)				
Yes	286 (44%)	70 (38%)	105 (38%)	111 (37%)
No	362 (56%)	114 (62%)	175 (62%)	188 (63%)
Comorbidities (*n* = 763)				
HIV	26 (3%)	7 (4%)	5 (2%)	14 (5%)
Malaria	79 (10%)	23 (13%)	26 (9%)	30 (10%)
Duration of symptoms prior to first hospital admission at MTRH (*n* = 739)				
≤ 1 month	116 (16%)	36 (31%)	41 (35%)	39 (34%)
1–3 months	318 (43%)	77 (24%)	124 (39%)	117 (37%)
> 3 months	305 (41%)	69 (23%)	115 (38%)	121 (40%)

**Fig. 2:**
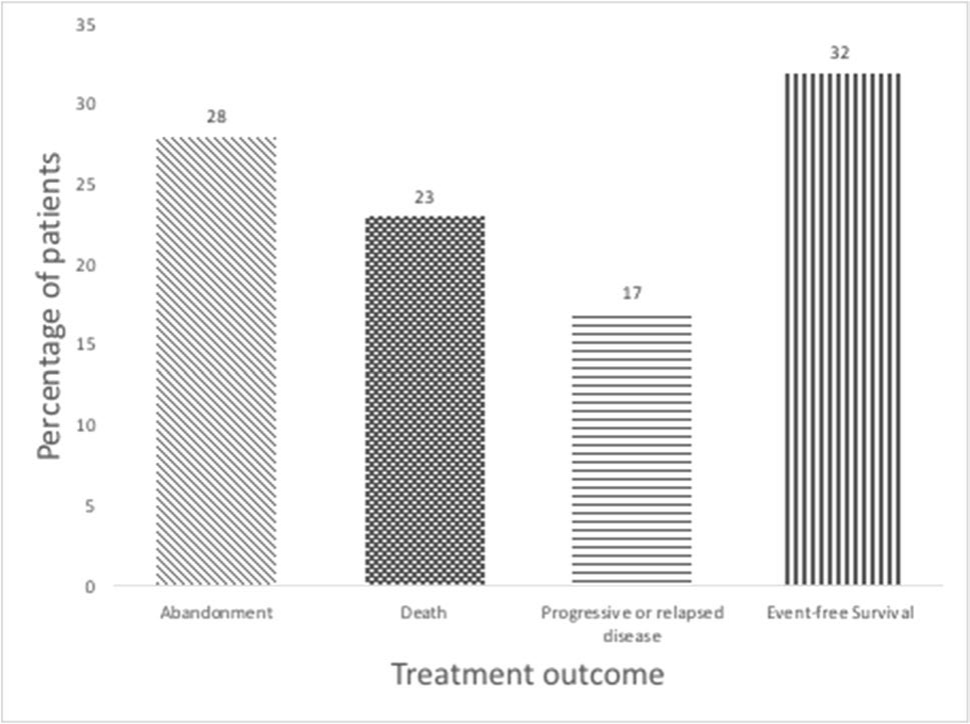
Treatment outcomes of children diagnosed with cancer at MTRH between January 2010 and December 2016 (*n* = 763)

**Fig. 3:**
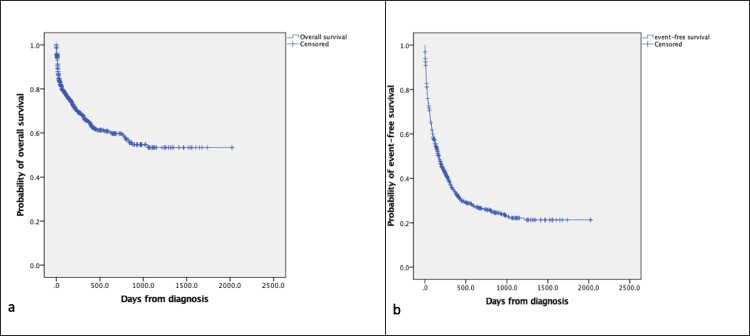
Kaplan–Meier estimates of overall survival (panel **a**) and event-free survival (panel **b**) in children diagnosed with cancer at MTRH between January 2010 and December 2016 (*n* = 763). Events included abandonment of treatment, death and progressive or relapsed disease

Hence, a total of 763 children were included for further analysis. The most common cause of treatment failure among these children was abandonment of treatment, as presented in Fig. 2. In total, 212 patients (28%) abandoned treatment, of which 14 (7%) dropped-out before start of treatment and 198 (93%) during treatment. The second most common cause of treatment failure was death (*n* = 178, 23%), of which 19 patients (11%) died before, 150 (83%) during treatment and 9 (6%) after completion of treatment. The least common cause of treatment failure was progressive or relapsed disease (*n* = 129, 17%), of which 68 patients had progressive disease and 61 had relapsed disease. In summary, 28% abandoned treatment, 23% died and 17% had progressive or relapsed disease resulting in 32% event-free survival. Figure 3 shows the overall survival and event-free survival of all 763 patients. Note that Fig. 2 shows actual percentages, whereas the Kaplan–Meier estimates in Fig. 3 shows time-dependent probability estimates.

**Fig. 4:**
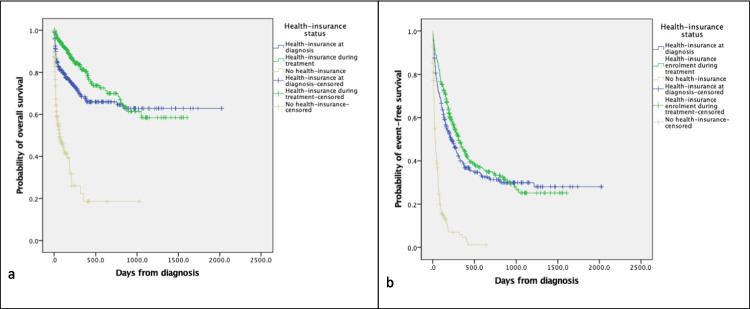
Kaplan–Meier estimates of overall survival (*P* < 0.001) (panel **a**) and event-free survival (*P* < 0.001) (panel **b**) in children diagnosed with cancer at MTRH between January 2010 and December 2016 per health-insurance status. Events included abandonment of treatment, death and progressive or relapsed disease

Among the 763 patients, 280 patients (37%) had NHIF coverage at diagnosis and 483 patients (63%) did not. Of the 483 patients without NHIF coverage, 299 patients (62%) enrolled during cancer treatment at MTRH, leading to a total NHIF registration level of 579 patients (76%). No significant differences in gender, age and type of cancer were found in children with different NHIF status. Table 2 shows that the treatment outcomes of patients differ per health-insurance status (*P* < 0.001). The most likely treatment outcome in uninsured patients was death (49%), whereas in those with health-insurance at diagnosis and those who enrolled during treatment it was event-free survival (36% and 41% respectively). Figure 4 shows that the overall survival (*P* < 0.001) and event-free survival (*P* < 0.001) were significantly higher in insured patients than in uninsured patients. Table 3 shows that several variables were found to be significant predictors of the hazard of death both in the unadjusted and adjusted models. Hazard-ratio for treatment failure was 0.30 (95% CI: 0.22–0.39; *P* < 0.001) for patients insured at diagnosis in relation to those without health-insurance and 0.32 (95% CI: 0.24–0.41; *P* < 0.001) for patients insured during treatment in relation to those without insurance.

**Table 2:** Treatment outcome per health-insurance status in children diagnosed with cancer at MTRH between January 2010 and December 2016 (*n* = 763; *P* < 0.001)

	No health-insurance (*n* = 184)	Health-insurance at diagnosis (*n* = 280)	Health-insurance enrolment during treatment (*n* = 299)
Death	90 (49%)	58 (21%)	31 (10%)
Abandonment of treatment	57 (31%)	70 (25%)	85 (28%)
Progressive/relapsed disease	16 (9%)	52 (19%)	61 (20%)
Event-free survival	21 (11%)	100 (36%)	122 (41%)

*P < 0.001*

**Table 3:** Regression analysis to determine the association between patient characteristics and treatment failure in children diagnosed with cancer at MTRH between January 2010 and December 2016

Variable	Unadjusted model:		Adjusted model:	
	Hazard ratio (95% CI)	*P*-value	Hazard ratio (95% CI)	*P*-value
Gender: Female	1.23 (0.98–1.55)	0.067	1.25 (0.98–1.60)	0.074
Age at Diagnosis	1.07 (1.05–1.09)	< 0.001	1.07 (1.05–1.09)	< 0.001
Patients’ residence	0.86 (0.70–1.05)	0.137	0.83 (0.67–1.03)	0.089
Distance to hospital	1.18 (1.02–1.37)	0.027	1.1 (0.92–1.32)	0.291
Health-insurance status	0.77 (0.63–0.94)	0.010	0.82 (0.67–1.02)	0.072
Type of cancer: Solid tumours	1.24 (1.00–1.53)	0.049	1.24 (1.00–1.53)	0.050
Type of cancer: Neuro-oncology tumors	1.78 (1.34–2.35)	< 0.001	1.8 (1.35–2.40)	< 0.001
Duration of symptoms	1.09 (1.06–1.12)	< 0.001	1.07 (1.04–1.10)	< 0.001
Referred from other facility	1.32 (0.92–1.90)	0.129	1.08 (0.74–1.58)	0.693
Diagnosed at other facility	1.11 (0.91–1.36)	0.298	1.08 (0.88–1.33)	0.470

The evaluation of other social and clinical characteristics revealed that factors such as age, gender, type of cancer, distance to MTRH, duration of symptoms prior to first hospital admission at MTRH, and being referred or diagnosed at other facilities did not have a significant statistical effect on the treatment outcomes.

## Discussion

This study highlights the survival of one of the largest cohorts of children treated for cancer in a single centre in sub-Saharan Africa. Childhood cancer event-free survival rate in Western Kenya was 32%. Reasons for low survival rates of childhood cancer in low and middle-income countries are multiple and interrelated. Many children have no access to healthcare and remain undiagnosed, and those with access to healthcare are either misdiagnosed or have delayed diagnosis thus presenting with advanced stage of disease lowering the probability of cure. Other causes include abandonment of treatment, death from toxicity, higher rates of relapse, limited multidisciplinary staff, sporadic supply of chemotherapy, and a limited number of centres that provide comprehensive cancer treatment [5, 22, 23].

The reported event-free survival rate of 32% is an increment from the previous rate of 19% for the years 2007–2009 in the same centre [21]. The improved survival can be attributed to adoption of standardized treatment protocols, reduction in treatment abandonment, better multidisciplinary care and more knowledgeable health personnel.

This study indicates that the two most commonly diagnosed childhood cancers were non-Hodgkin’s lymphoma and acute lymphoblastic leukaemia followed closely by nephroblastoma. This is different from an earlier study done in the same hospital between the years 2006–2010 where non-Hodgkin’s lymphoma was by far the most common diagnosis [20]. Non-Hodgkin’s lymphoma presents as a swelling that is easily recognizable therefore most of these patients were already getting to hospital. The proportionate increase in the number of childhood cancers diagnosed could be explained by increased awareness among health care workers, better equipping of hospitals allowing more hemograms and ultrasounds to be done and improved referral pathways. The current distribution is closer to what is seen in high-income countries except for the very low numbers of brain tumours which are the second commonest childhood cancers diagnosed in high-income countries [20, 24]. The low numbers of brain tumours can be attributed to the difficulty in diagnosis which requires imaging with CT scan or MRI which were not readily available and low index of suspicion among health care workers [20].

The leading cause of treatment failure in our study was treatment abandonment. This is similar to other studies in low and middle-income countries where it contributes 50–60% of treatment failure. This is in contrast to high-income countries where toxicity-related death and relapse are the most common causes of treatment failure. Abandonment has been linked to poverty, treatment costs, access to health-insurance, transport costs, loss of income, inadequate counselling, use of traditional and complementary medicine and parents’ lack of hope for their child’s cure [22, 23]. In our centre, the most common reasons for treatment abandonment were financial difficulties and inadequate access to NHIF [22]. The treatment abandonment rate of 28% in this study is lower than the 54% rate reported in our centre for children who were diagnosed in the years 2007–2009 [21]. This change can partially be attributed to efforts of dedicated personnel assisting families to register for the national health-insurance, enhanced counselling to parents, and follow-up phone calls to those who have missed their hospital appointment. In Latin-America, centres have tried to reduce treatment abandonment by improving record-keeping, patient-tracking, psychological support and financial assistance to families which has led to a reduction of treatment abandonment from 15% to less than 3% [25]. Prevention of abandonment is as important as prevention of treatment-related mortality or relapse and should be a priority for clinicians and policy makers [26].

Cancer treatment completion is dependent on the ability to cater for the costs of treatment through personal finances or health-insurance. In many sub-Saharan African countries, access to health-insurance is limited and many families are of poor socio-economic status. Even with the availability of treatment, financial unaffordability forces many children in low and middle-income countries to abandon treatment. Individuals who do not have access to health-insurance are more likely to be diagnosed with late-stage cancer resulting in markedly worse outcomes [10, 11, 13, 27]. This study demonstrates that not having insurance led to worse outcomes compared to those who had it at diagnosis or enrolled and became beneficiaries of health-insurance during the course of treatment. Therefore, in order to improve treatment outcomes, it is important for healthcare providers to actively help families with health-insurance registration. In an effort to increase health-insurance coverage, the government could make it mandatory whereby anyone with an income should pay and the most vulnerable in the society should be supported by the government.

Majority of childhood cancer deaths occur in low and middle-income countries where there is often poor access to health services. It is crucial that as countries progress to UHC, childhood cancers are included in benefits packages. Distribution of resources for cancer control lacks equity and low and middle-income countries have less than 5% of global resources for cancer care and control. There is a need to invest in the scale-up of diagnosis, treatment, and care of children with cancer in order to address the unacceptable inequalities in access to healthcare and health outcomes within and between countries and prevent needless deaths in children [27, 28].

In 2005, an international plea was made by the United Nations, World Health Organization and over 500 health and development organizations for UHC. Although implementation of this health finance system ensures that all people have access to services and not suffer financial hardship, only a few governments of low and middle-income countries have complied. UHC was included as one of the four big priorities of the sustainable development agenda by the Kenyan government in 2018 and is in pursuance of the human right to health which is enshrined in the country’s 2010 Constitution [1, 3, 29]. NHIF introduced reforms in 2015 in a bid to attain UHC by revising the premium contribution rates upwards and in turn expanded its benefit packages to include an oncology package that caters for chemotherapy and radiotherapy [30]. This additional package by NHIF is the right direction towards ensuring that families do not suffer financial adversity while seeking medical services. What is required now is to ensure that more families are enrolled into the scheme.

The main limitations of this study were missing medical records and incomplete or lack of documented data due to the retrospective design. Substantial effort was required to retrieve medical files and extract reliable information. In total 116 records were lost. The data of these children is therefore absent from the analyses, which may influence the outcomes found although no selection bias was shown. Therefore, documentation of all clinical characteristics should be intensified as well as ensuring that medical records are kept well.

In conclusion, survival of children diagnosed with cancer between 2010 and 2016 in a Kenyan tertiary teaching hospital was found to be 32%, significantly higher than in an earlier report. The most common cause of treatment failure was abandonment of treatment followed by death. Patients who had health-insurance had a much higher chance of survival than those without. In order to increase survival, we therefore need to put in measures to reduce abandonment and increase health-insurance coverage. Some of the measures to reduce abandonment would include parental education, giving families social support through parental support groups and financial assistance from the government or philanthropic organizations. In order to increase health-insurance coverage, the government should make it mandatory and also sensitize the public on the benefits of health-insurance. UHC should be integrated into national health strategies since this will ultimately lead to better childhood cancer survival.

## Data Availability

Data can be madeavailable upon reasonable request.

## Abbreviations

UHC: Universal Health Coverage
NHIF: National Hospital Insurance Fund
